# This Article Corrects: “The Bullets He Carried”

**DOI:** 10.5811/westjem.69717

**Published:** 2026-11-02

**Authors:** Stephen Hargarten

**Affiliations:** Medical College of Wisconsin, Comprehensive Injury Center, Milwaukee, Wisconsin

*Original article:* doi 10.5811/westjem.2020.5.48216

*Original title:* The Bullets He Carried

Author: Stephen Hargarten, MD, MPH

Wound Ballistics Testing Update: A Revision to the September 2020 Commentary

Since my September 2020 Commentary was published,^1^ we have expanded our wound ballistics research by adding a testing site at Cranfield Defense and Security in England. This partnership was established to replicate our findings from the Milwaukee site and explore additional firearms, including historical black powder weapons.

Ballistics tests at Cranfield were conducted using a muzzle-loading flintlock Brown Bess musket (circa 1780s) firing a 41-g musket ball, adhering to their standardized black powder protocol. (The initial simulation model used a musket ball weighing less than 4 g.). Kinetic energy transfer tests with this heavier musket ball averaged 524 J, using the same 20% gelatin matrix employed across both research sites. Additionally, pooled testing data for the AR-15 (5.56-mm NATO round) were updated and summarized, yielding an average kinetic energy transfer of 1,363 J, compared with the 1055.5 J initially reported.

To update the comparative findings from the original commentary, the baseline rate of fire and historical equivalents remain unchanged: The musket rate of fire is 2 rounds/min, and the perpetrator’s rate of fire during the Sandy Hook Elementary School shooting remains 39 rounds/min—a volume of fire that historically represents 19 militia members storming the school. However, integrating the updated kinetic energy measurements alters the total energy-transfer-per-minute calculations as follows:

- Musket: 2 rounds/min × 524 J = 1048 J/min
- AR-15: 39 rounds/min × 1363 J = 53,157 J/min

This updated calculation reveals that the kinetic energy potential of the shooter represents 51 militia members storming the school simultaneously. This finding marks a significant downward revision from the 171 militia members calculated using the initial, lighter simulation ball.

We continue our rigorous wound ballistics testing and welcome critical peer review from the readership to ensure accurate, replicable, and scientifically sound data.
